# Gentiopicroside injection promotes the healing of pressure injury wounds by upregulating the expression of bFGFR1

**DOI:** 10.1590/1980-220X-REEUSP-2023-0183en

**Published:** 2024-07-08

**Authors:** Qiang Li, Xiaoshuan Liu, Min Zhang, Jungang Liu, Juan Lu

**Affiliations:** 1 Gansu Provincial Hospital of Tradicional Chinese Medicine, Lanzhou, Gansu, China.

**Keywords:** Pressure Injury, Gentianaceae, Receptor, Fibroblast Growth Factor, Type 1, Proliferating Cell Nuclear Antigen, Wound Healing, Úlcera por Pressão, Gentianaceae, Receptor Tipo 1 de Fator de Crescimento de Fibroblastos, Antígeno Nuclear de Célula em Proliferação, Cicatrização, Úlcera por Presión, Gentianaceae, Receptor Tipo 1 de Factor de Crecimiento de Fibroblastos, Antígeno Nuclear de Célula en Proliferación, Cicatrización de Heridas

## Abstract

**Objective:**

To observe the therapeutic effect of gentiopicroside, as the main component of Gentianaceae, on wounds in pressure injury (PI) model rats and explore its mechanism.

**Method:**

Male Sprague Dawley rats were randomly divided into control group, model group and gentiopicroside groups (50, 100 and 200 mg·kg^-1^·d^-1^ for 9 consecutive days). The mice’s skeletal muscle fibroblast line NOR-10 cells were collected after being treated with gentiopicroside (0.2~5.0 M) and basic fibroblast growth factor receptor 1 (bFGFR1) inhibitor (5.0 M SU5402) for 7 days.

**Results:**

Compared to the model group, the gentiopicroside groups showed significantly increased wound healing rates, reduced inflammatory cells in the wound tissues, and significantly increased expression levels of proliferating cell nuclear antigen (PCNA) and bFGFR1, accompanied by increased proliferation of new myofibroblasts. Gentiopicroside upregulated the mRNA expression of bFGFR1 and PCNA in NOR-10 cells in a dose-dependent manner; however, SU5402 reversed the effect of gentiopicroside.

**Conclusion:**

Gentiopicroside may promote myofibroblast proliferation by upregulating the expression of bFGFR1 and PCNA and ultimately accelerating the healing of PI wounds.

## RESUMO

**Objetivo:**

Observar o efeito terapêutico do gentiopicrosídeo como principal componente das Gentianáceas em feridas de lesão por pressão (LP) em modelos de ratos e explorar seu mecanismo.

**Métodos:**

Ratos Sprague Dawley machos foram divididos aleatoriamente em grupo controle, grupo modelo e grupos gentiopicrosídeo (50, 100 e 200 mg·kg^-1^·d^-1^ por 9 dias consecutivos). As células NOR-10 da linha de fibroblastos do músculo esquelético de camundongos foram coletadas após serem tratadas com gentiopicrosídeo (0,2~5,0 μM) e inibidor do receptor 1 do fator de crescimento fibroblástico básico (bFGFR1) (5.0 μM SU5402) por 7 dias.

**Resultados:**

Em comparação com o grupo modelo, os grupos gentiopicrosídeo apresentaram taxas de cicatrização de feridas significativamente maiores, menos células inflamatórias nos tecidos da ferida e níveis de expressão de antígeno nuclear de proliferação celular (PCNA) e bFGFR1 significativamente maiores, acompanhados por aumento da proliferação de novos miofibroblastos. O gentiopicrosídeo regulou positivamente a expressão de mRNA de bFGFR1 e PCNA em células NOR-10 de maneira dependente da dose, enquanto o SU5402 reverteu o efeito do gentiopicrosídeo.

**Conclusão:**

O gentiopicrosídeo pode promover a proliferação de miofibroblastos, suprarregulando a expressão de bFGFR1 e PCNA e, em última análise, acelerando a cicatrização de feridas de LP.

## RESUMEN

**Objetivo:**

Observar el efecto terapéutico del gentiopicrósido como componente principal de la Gentianaceae en heridas por lesión por presión (LP) en modelos de ratas y explorar su mecanismo.

**Método:**

Se dividieron aleatoriamente ratas macho Sprague Dawley en grupo control, grupo modelo y grupos gentiopicrósido (50, 100 y 200 mg·kg^-1^·d^-1^ durante 9 días consecutivos). Se recogieron células NOR-10 de la línea de fibroblastos de músculo esquelético de ratón después de ser tratadas con gentiopicrósido (0.2~5.0 μM) y un inhibidor del receptor 1 del factor de crecimiento de fibroblastos básico (bFGFR1) (5.0 μM SU5402) durante 7 días.

**Resultados:**

En comparación con el grupo modelo, los grupos de gentiopicrósido mostraron tasas de curación de heridas significativamente más altas, menos células inflamatorias en los tejidos de la herida y niveles de expresión significativamente mayores del antígeno nuclear de proliferación celular (PCNA) y bFGFR1, acompañados de una mayor proliferación de nuevos miofibroblastos. El gentiopicrósido podría regular positivamente la expresión de ARNm de bFGFR1 y PCNA en células NOR-10 de manera dependiente de la dosis, sin embargo, SU5402 revirtió el efecto del gentiopicrósido.

**Conclusión:**

El gentiopicrósido puede promover la proliferación de miofibroblastos al aumentar la expresión de bFGFR1 y PCNA y, en última instancia, acelerar la cicatrización de las heridas de LP.

## INTRODUCTION

Pressure injury (PI) commonly occurs in association with long-term clinical bed rest or fractures. It tends to occur in the parts of bone, joint protrusions, and fragile soft tissue under prolonged pressure. PI includes tissue ulceration and necrosis caused by blood circulation disorders, cell ischaemia and hypoxia, or nutrient metabolism disorders in local tissues. Debridement and dressing changes are often used for the clinical treatment of PI, which has clinical characteristics such as a delayed course of disease, easy recurrence, and difficult recovery and is identified as a difficult surgical disease by the National Administration of Traditional Chinese Medicine^(1,2)^. Severe PI can cause flesh rot and bone exposure and infection and can even lead to sepsis, endangering the life of patients because of its high complications and mortality^(1-4)^. At the same time, PI increases the workload of nursing staff and the economic burden of medical treatment. Therefore, seeking effective treatment measures for PI has always been a focus of medical attention.

Gentiopicroside (GPS, PubChem CID: 8708) is the main active component of gentianaceae plants and is a schizocyclic iridoid glycoside, an easily accessible and low-cost product. It has been reported to be effective for fatty liver, arthritis, colitis, diabetic peripheral neuralgia and other diseases because of its anti-inflammatory, swelling reduction, analgesic, and antiviral properties, liver and gallbladder protection, and other effects^(5-11)^. The tissue repair effect of GPS may be achieved by inhibiting the inflammatory signaling pathway NF-κB, reducing the levels of inflammatory mediators in blood and tissues, increasing blood flow velocity, and so on^(5-11)^. Therefore, GPS may be an effective drug for the treatment of PI. In this study, the efficacy of GPS in the treatment of PI was examined, and its mechanism was explored through animal and cell experiments.

## METHOD

### Study Design

This pre-clinical study investigated the effect of GPS on PI and explored its mechanism through both a PI animal model and the mouse skeletal muscle fibroblast line NOR-10.

SPF male Sprague Dawley rats weighing 180-220 g (animal licence No. SCXK (Shanxi, Chima) 2018-001, Experimental Animal Facilities Licence No. SYXK (Gansu, China) 2011-0001) were raised in the Animal Experiment Center of Gansu University of Traditional Chinese Medicine at room temperature (18-22 °C) and humidity of 40-60%, with food ad libitum. After 1 week of adaptive feeding, the rats were randomly divided into a normal control group (Control group), a model control group (Model group) and experimental groups respectively receiving low, medium and high dose of GPS (50, 100, 200 mg·kg^–1^·d^–1^) (n = 12 per group except the control group). Eight rats from each group of Control group, Model group and GPS treatment groups were sacrificed to detect the mRNA and protein levels of PCNA, bFGFR1 and HE staining, and the other four rats from each group of model group and GPS treatment groups were observed of wound complete-healing time.

### PL Model

Except for the normal control group, the rats in each group were subjected to PI modelling according to the literature^(12)^. During the experiment, rats were anaesthetized by intraperitoneal injection of pentobarbital sodium (0.1 mL, 10 mg/kg). First, the skin on the back of the rat’s buttocks was sterilized, an incision approximately 2 cm long was made in the skin of the buttocks, and then the gluteus maximus was passively separated with surgical tweezers to implant a magnetic disc with a diameter of 15 mm and a thickness of 2 mm into the gluteus maximus. The skin was sutured and sterilized. After 24 hours, another magnet was placed on the skin above the implanted magnetic disc. The two magnets produced magnetic force to cause local ischaemia. After 2 hours, the external magnet was removed, and the above steps were repeated after 20 minutes, 4 times a day, for 4 consecutive days. The criteria for successful PI modelling were as follows: the skin under pressure becomes black and hard, and does not bleed in response to a pinprick.

### Drugs

Analytically pure GPS was purchased from Sichuan Heyikang Biotechnology Co., Ltd. (20831-76-9). In animal experiments, GPS was dissolved in normal saline to prepare a stock solution with a final concentration of 400 mg·ml^-1^. In cell experiments, GPS was dissolved in Dimethyl sulfoxide (DMEM) to prepare a stock solution with a final concentration of 5 mM, which was stored in a refrigerator at 4 °C for later use.

### Experimental Animals and Treatment

From the day of modelling, the rats of GPS groups were given different doses of GPS solution (50, 100, 200 mg·kg^–1^·d^–1^) by intramuscular injection once a day for 9 consecutive days, and the dosage selected was based on our previous experimental results and literature^(13)^. The wound area and healing rate of each group were observed on the 3rd, 5th, 7th and 9th days. On the 9th day, 8 rats in each group were randomly selected to be killed under anaesthesia, and the serum and wound tissue were collected. The other rats in the GPS groups continued to receive treatment until their wounds were cured.

### Cells and Treatment

The mouse skeletal muscle fibroblast line NOR-10 was purchased from Shanghai Jia He Biotechnology Co., Ltd. and cultured in DMEM containing 10% FBS at 37 °C and 5% CO_2_. NOR-10 cells were treated with 0.2~5.0 μM GPS and basic fibroblast growth factor receptor 1 (bFGFR1) inhibitor SU5402 (5.0 μM) (215543-92-3, Shanghai Lanmu Chemical Co., Ltd.). The drug-containing culture medium was changed every day for 9 days, and the cells were collected.

### Wound Area, Healing Rate and Healing Time

On the 3rd, 5th, 7th, and 9th days after model construction, the maximum diameter of the PI wound was measured, and the wound area and healing rate of each group were calculated on the 9th day. Observation was continued until the wound was completely healed. Wound healing rate = (original area of wound - area of unhealed wound)/original area of wound × 100%.

### Data Collection

The muscle tissue in the compression area was cut along the direction of the muscle flap, and the cells were collected by trypsin digestion. The histomorphology of muscle tissue was observed by haematoxylin and eosin (H&E) staining. The mRNA expression levels of tissue proliferating cell nuclear antigen (PCNA) and bFGFR1 in tissues and cells were measured by qRT‒PCR. The protein expression levels of PCNA and FGFR1 were determined by immunohistochemistry.

### Pathophysiology

On the 9th day after model construction, the rats in each group were sacrificed under anaesthesia, the muscle tissue at the injection site was cut from the normal control group, and the muscle tissue at the PI wound site was cut from the rats in the other groups. The tissue was fixed in 10% formalin and then embedded in paraffin. Sectioning was performed at a thickness of 4 μm, routine HE staining was performed, and the morphology of the tissues was observed under an optical microscope.

### Immunohistochemistry

On the 9th day after model construction, the muscle tissue was fixed in 4% paraformaldehyde and rinsed with PBS, followed by antigen retrieval, elimination of endogenous peroxidase, blocking, and incubation with PCNA monoclonal antibody (BM0104, 1:400, Boster Company, USA)/anti-FGFR1 antibody (abs131707, 1:200, Absin Company, China) and pronase-HRP, DAB colouration, haematoxylin redyeing, transparency and sealing. Optical microscopy was performed to observe and photograph the tissue. Nikon image analysis software was used to calculate the total tissue area, positive staining area, and integrated optical density on each section, and the average grey value was calculated. Grey value = positive staining area/total tissue area × integrated optical density.

### Real-Time Quantitative PCR (RT–QPCR)

Total RNA was extracted from tissues and cells by TRIzol reagent. RNA concentration and purity were measured by a Nanodrop instrument, and the A260/A280 ratio was 1.8–2.1, indicating that the RNA quality was acceptable. According to the kit (Dalian Bao Bioengineering Co., Ltd., China) instructions, the RNA was reverse transcribed into cDNA. The primer sequences (Tianyi Huiyuan Biotechnology Co., Ltd., China) are as follows: upstream primer for GAPDH was 5’-GCAAGTTCAACGGCACAG-3’, downstream primer was 5’-GCCAGTAGACTCCACGACA-3’; the upstream primer for bFGFR1 was 5’-CAGGGCTACCAGCCAACAA-3’, the downstream primer was 5’-CACTGTACACCTTGCACATGAACTC-3’; the upstream primer for PCNA was 5’-TTGGCAATGGGAACA-3’; and downstream primer was 5’-GACAGTGGAGTGGCTTT-3’. RT‒qPCR was then performed using a SYBR Green Quantitative PCR Master Mix Kit and ABI StepOnePlus Cycler (Thermo Fisher Scientific, Waltham, MA, USA), with an initial denaturation step at 95°C for 5 min. Each reaction was then cycled 40 times, with each cycle consisting of 95°C for 5 s, followed by an annealing step, and a final elongation step at 72°C for 30 s if the annealing temperature was below 60°C. The relative expression of each gene was calculated using the ΔΔCt method with GAPDH as an internal reference.

### Ethical Aspects

SPF male SD rats were purchased from the Laboratory Animal Center of Xi’an Jiaotong University School of Medicine, Animal Licence No. SCXK (Shan) 2018-001, and were raised in the Animal Experimental Center of Gansu University of Traditional Chinese Medicine, Experimental Animal Facility Licence No. SYXK (Gansu) 2011-0001. All procedures performed in this study were in accordance with the Ethical Principles of Animal Experiments adopted by the Medical Ethics Committee of the 940th Hospital of Joint Logistics Support force of Chinese People’s Liberation Army (Approval number: 2020KYLL071).

### Statistical Analysis

SPSS 22.0 software was used to process the data. Graph Pad Prism 5 was used to analyse the data, and the quantitative data are expressed as the mean ± S.E.M. The F-test was used in the comparison among the treatment groups and the t-test was used for comparisons between two groups. The data from cell experiments with different drug concentrations were analysed using one-way ANOVA. Statistical significance was defined as *P* < 0.05.

## RESULTS

### Wound Healing Area, Complete-Healing Time and Healing Efficiency

**Figure 1:**
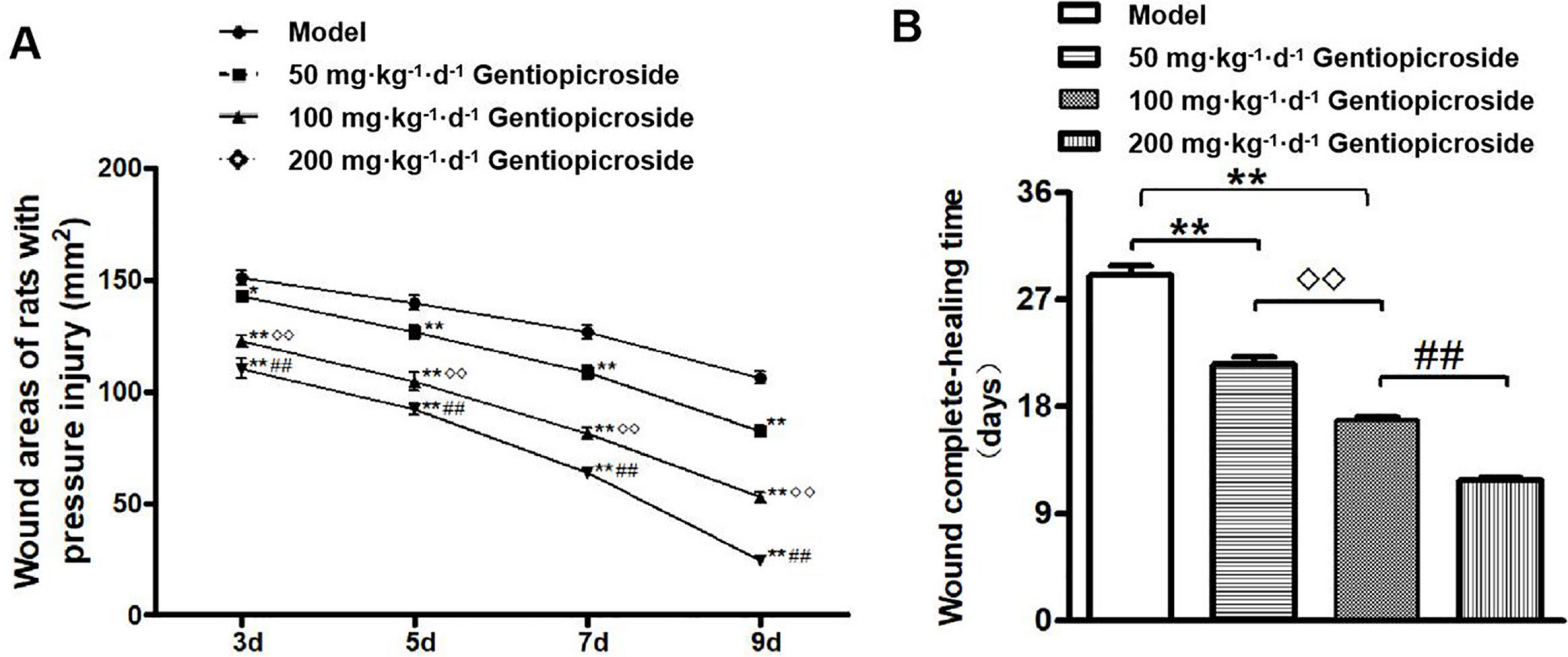
Wound area and complete-healing time of pressure injury rats. A. Wound areas of rats with pressure injury (n = 12 per group); B. Wound complete-healing time (n = 4 per group). Mean ± S.E.M. *P < 0.05, **P < 0.01 vs. Model; ^◊◊^P < 0.01 vs. 50 mg·kg^–1^·d^–1^ GPS; ^#^P < 0.05, ^##^P < 0.01 vs. 100 mg·kg^–1^·d^–1^ GPS. – Gansu, China, 2020.

At 3, 5, 7 and 9 days after drug treatment in SD rats, the PI area of rats in each GPS group was significantly smaller than that in the model group (*P* < 0.05), and the amount of traumatic exudate, redness and swelling and neonatal granulation tissue were all better. The 200 mg·kg^–1^·d^–1^ GPS group showed the most significant effect (*P* < 0.01). After 9 days of drug treatment, the trauma of rats in the 200 mg·kg^–1^·d^–1^ GPS group was basically healed, although some rats had areas of unhealed wounds. No rats in the model group were completely healed. The trauma area of each group is shown in Figure 1A. Compared with that in the model group, the wound complete-healing time was shortened in the GPS groups with all doses (*P* < 0.01), as shown in Figure 1B. The PI wound healing rate in each GPS group at different doses was higher than that of the model group (*P* < 0.05), and difference in the 200 mg·kg^–1^·d^–1^ GPS group was significant using the strictest threshold (*P* < 0.01), as shown in Table 1.

**Table 1:** The average percentage of wound healing of rats with pressure injury – Gansu, China, 2020.

Group	Dose of gentiopicroside (mg·kg^-1^·d^-1^ for 9 days)	The average percentage of healing (%)			
		3d	5d	7d	9d
Model	0	14.67 ± 1.86	20.93 ± 1.81	28.40 ± 1.67	39.94 ± 1.66
Low dose	50	19.23 ± 1.87*	28.32 ± 3.41**	38.62 ± 3.69**	53.45 ± 2.95**
Medium dose	100	30.59 ± 2.79** ^◊^	40.85 ± 2.43** ^◊^	53.98 ± 1.26** ^◊^	70.12 ± 1.55** ^◊^
High dose	200	37.60 ± 3.01**^#^	48.01 ± 2.08**^#^	64.09 ± 3.32**^##^	86.08 ± 2.32**^##^

n = 12 per group; Mean ± S.E.M.

*P < 0.05,

**P < 0.01 vs. Model;

^◊^P < 0.01 vs. Low dose;

^#^P < 0.05,

^##^P < 0.01 vs. Medium dose.

### He Staining Results of Wounded Myofibroblastic Tissue

**Figure 2:**
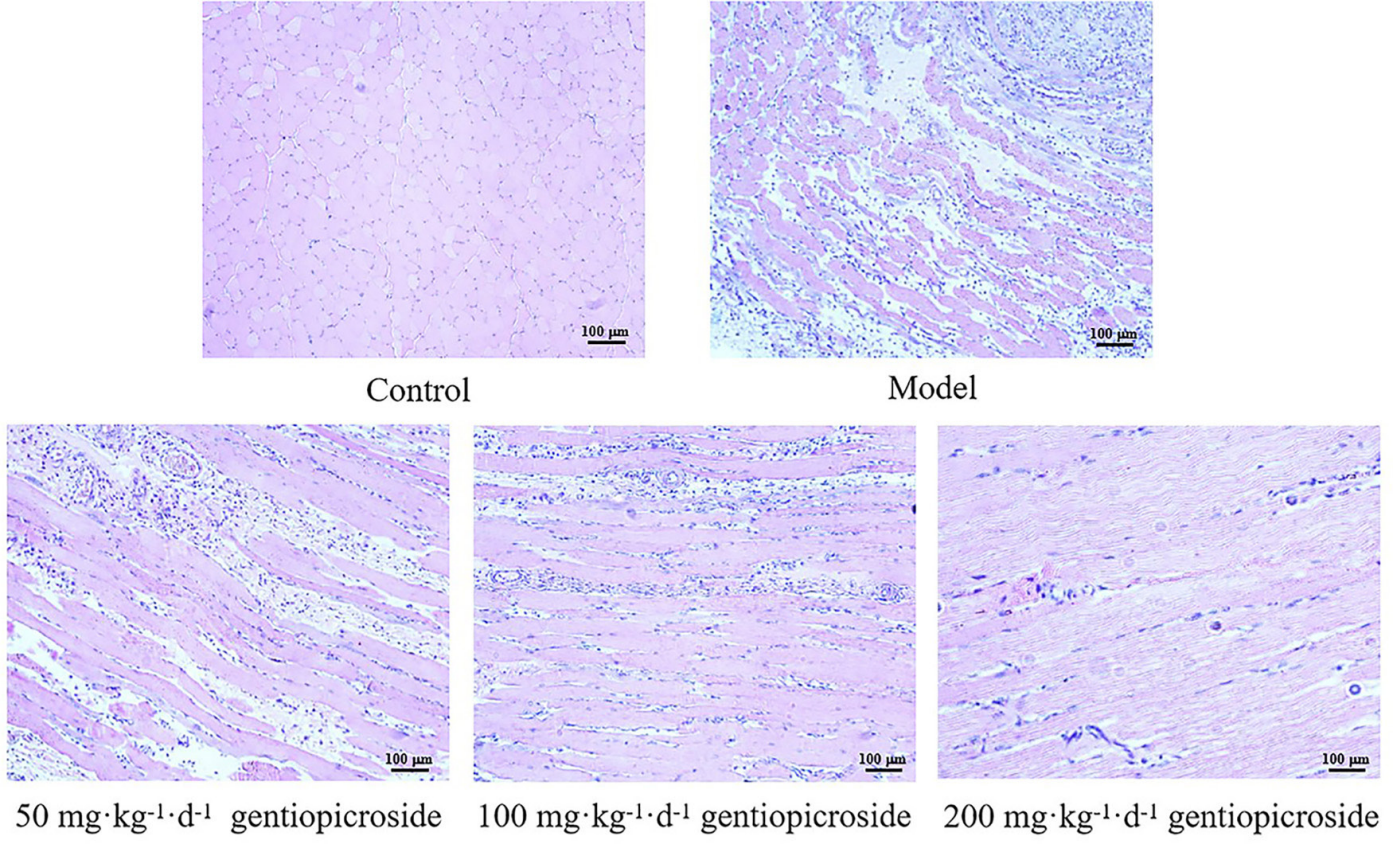
HE staining of myofibroblasts in the wound surface of rats. n = 8 per group – Gansu, China, 2020.

Nine days after model construction, as shown in Figure 2, the results of HE staining showed that the control group had a clear tissue structure. Large amounts of inflammatory cell infiltration were observed in the injured tissues of the model group, accompanied by tissue oedema and vasodilation. In the 50 mg·kg^–1^·d^–1^ GPS group, more inflammatory cell infiltration, tissue oedema, and vasodilation were observed. A small amount of inflammatory cell infiltration was observed in the 100 mg·kg^–1^·d^–1^ GPS group. Sporadic inflammatory cells were observed in the 200 mg·kg^–1^·d^–1^ GPS group, with no vasodilation and more myofibroblast proliferation.

### PCNA and FGFR1 Expression Levels in Traumatic Myofibroblastic Tissue

**Figure 3:**
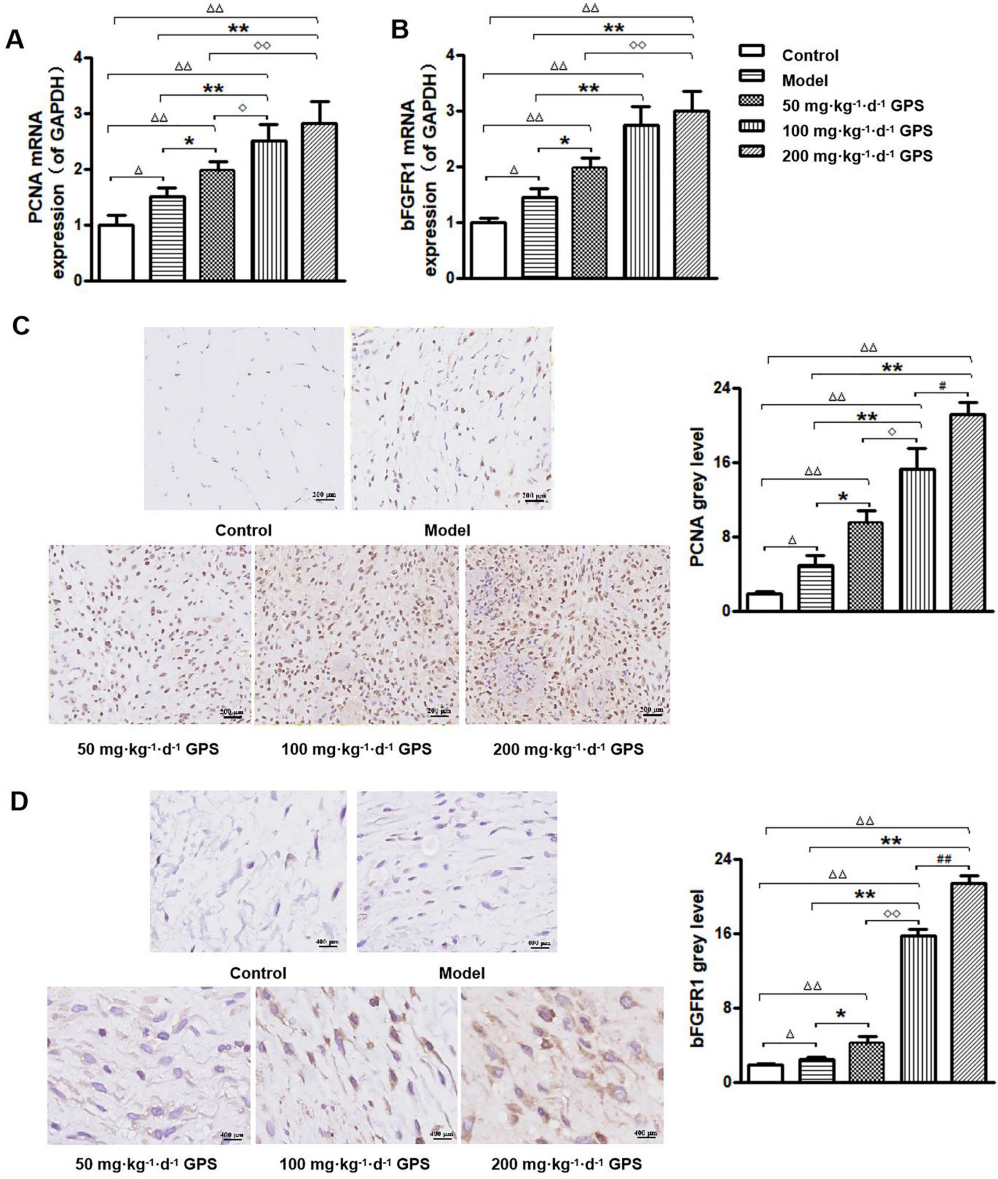
Expression levels of proliferating cell nuclear antigen (PCNA) and basic fibroblast growth factor receptor 1 (bFGFR1) in the myofibroblast tissue of the rat wound (n = 8 per group). A–B PCNA and bFGFR1 mRNA expression levels; C-D PCNA and bFGFR1 protein expression levels. GPS, Gentiopicroside. Mean ± S.E.M., ^∆^P < 0.05,^∆∆^P < 0.01 vs. Control; *P < 0.05, **P < 0.01 vs. Model; ^◊^P < 0.05, ^◊◊^P < 0.01 vs. 50 mg·kg^–1^·d^–1^ Gentiopicroside (GPS); ^#^P < 0.05, ^##^P < 0.01 vs. 100 mg·kg^–1^·d^–1^ GPS – Gansu, China, 2020.

Nine days after model construction, RT‒PCR results showed that compared with that in the control group and model group, the mRNA expression of PCNA and bFGFR1 in the GPS group was significantly upregulated (*P* < 0.05), and the expression levels in 200 mg·kg^-1^·d^-1^ GPS group were the highest (*P* < 0.01) (Figure 3A–B). The immunohistochemistry results showed that there were almost no myofibroblasts or PCNA-positive cells in the control group. Some myofibroblasts and PCNA-positive cells were observed in the model group. Myofibroblast proliferation and PCNA-positive cell distribution were observed to be increased in each GPS group, especially in the 200 mg·kg^–1^·d^–1^ GPS group (*P* < 0.01) (see Figure 3C). In addition, compared to that in the control group and the model group, the expression of bFGFR1 in the GPS groups was significantly upregulated (*P* < 0.05), and the 200 mg·kg^–1^·d^–1^ GPS group was the most significant (*P* < 0.01), as shown in Figure 3D.

### Effects of GPS on BFGFR1 and PCNA Expression in NOR-10 Cells

**Figure 4:**
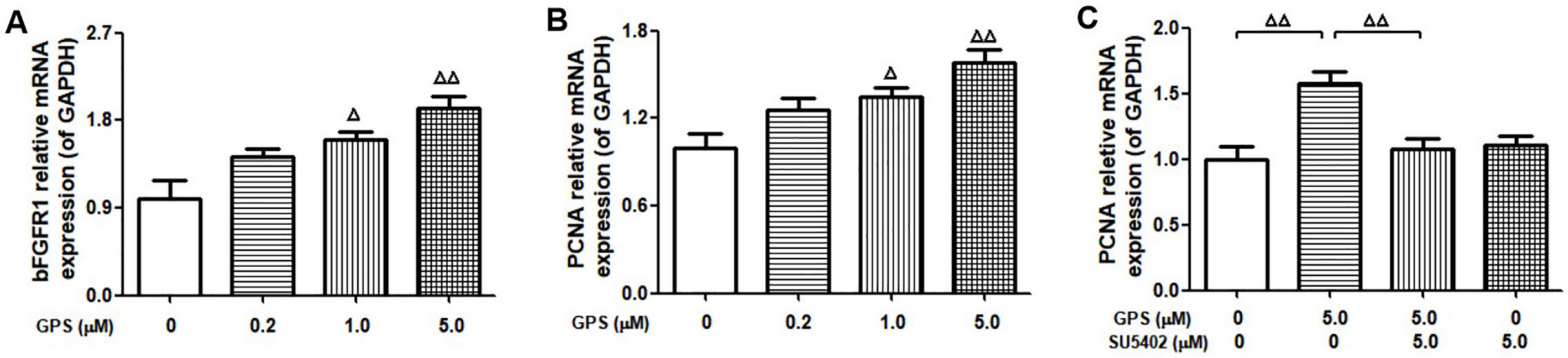
Effects of gentiopicroside (0.2~5.0 μM GPS) and basic fibroblast growth factor receptor 1 (bFGFR1) inhibitor (5.0 μM SU5402) on bFGFR1 and proliferating cell nuclear antigen (PCNA) expression in NOR-10 cells (n = 3). ^∆^P < 0.05, ^∆∆^P < 0.01 vs. control – Gansu, China, 2021.

NOR-10 cells were treated with 0.2~5.0 μM GPS for 7 days. Both bFGFR1 and PCNA mRNA expression showed significant dose-dependent increases (*P* < 0.05) (Figure 4A–B), and the bFGFR1 inhibitor SU5402 (5.0 μM) reversed the upregulation effect of PCNA by GPS (Figure 4C).

## DISCUSSION

Trauma usually causes varying degrees of cell degeneration, necrosis and tissue defects. Cell proliferation plays a leading role in tissue repair, and the integrity and speed of trauma repair depend on the regenerative ability and proliferation rate of cells in the traumatized tissue. Because fibroblasts can synthesize and secrete dermal collagen, matrix proteins, growth factors, and cytokines, therapies targeting fibroblast proliferation may enable more effective treatment for PI^(14,15)^. Our animal experiment results showed that local intramuscular injection of GPS in PI model rats can promote wound healing, reduce the number of inflammatory cells in the wound area, which is consistent with the literature reports that GPS has anti-inflammatory effects (inhibiting inflammatory signaling pathways and reducing inflammatory mediators)^(5-11)^. More importantly, we also found that GPS can promote the proliferation of myofibroblasts, and ultimately significantly shorten the healing time, reduce the wound area, and improve the wound healing rate. Especially for deep tissue injury, our results showed that injection of GPS effectively promoted PI healing by penetrating into the deep tissue. These results suggest that GPS injections can accelerate the healing of deep wounds in PI, which may be related to the promotion of myofibroblast proliferation and the anti-inflammatory effect.

Further, we investigated the mechanism of GPS injection promoting myofibroblast proliferation. As a proliferating nuclear antigen, PCNA can bind to DNA polymerase-affiliated proteins, participates in DNA replication, plays an important role in the initiation of cell proliferation, and is a useful indicator of cell proliferation status^(16,17)^. Basic fibroblast growth factor (bFGF) is a class of peptides or proteins with biological activities such as cell proliferation and differentiation, produced by nonglandular cells in paracrine or autocrine signalling, that exist in very low concentrations in the body but have extensive and effective biological activity, which plays a key regulatory role in wound healing^(18,19)^. When bFGF binds to its receptor bFGFR1, it participates in various signal transduction pathways within the cell. Studies have reported that bFGF can upregulate PCNA expression in mouse spermatogonial stem cells and human retinoblastoma^(20-22)^, thereby regulating cell proliferation and differentiation^(22)^. Our animal results showed that the expression of bFGFR1 and PCNA in traumatized tissue from PI model rats increased after injection of GPS treatment, and cell experiments showed that the bFGFR1 inhibitor SU5402 could reverse the upregulation of GPS on PCNA in myofibroblasts. Based on the above results and literature, it was suggested that high expression of bFGFR1 mediates the upregulation of PCNA by GPS in myofibroblasts.

In reference to our previous study^(23)^, we have used the method of pressurizing steel column to make the mild PI modeling, and GPS powder was applied to the surface of wound. It was found that GPS powder had a therapeutic effect on PI, and could cause changes in mRNA and protein levels of gene PCNA, which is consistent with the conclusions of this paper and further confirms the therapeutic effect of GPS on PI. It has been reported that the p65 subunit of the transcription factor NF-κB may target the -348/-338 region of FGFR1 to promote FGFR1 transcription and enhance the pro-proliferation effect of FGFR1^(24)^. What’s more, transient receptor potential canonical 1(TRPC1) may be involved in bFGFR1 activation^(25)^. Whether the mechanism of upregulation of bFGFR1 by GPS is related to p65 and TRPC1 remains to be further explored.

## CONCLUSION

Based on the above findings, we believe that GPS may increase bFGF binding to bFGFR1 by upregulating the expression of bFGFR1 in PI tissue, thus inducing high expression of PCNA in myofibroblastsand consequently promoting the proliferation of myofibroblasts, meanwhile, GPS may reduce inflammatory cells by inhibiting inflammatory signaling pathways, ultimately accelerating PI healing.
